# Trait phenotyping and identification of trait-specific donor genotypes for agronomic improvement and ideotype breeding in browntop millet (*Urochloa ramosa* L.)

**DOI:** 10.3389/fpls.2026.1756143

**Published:** 2026-04-13

**Authors:** C. Nandini, Kommineni Jagadeesh, Dinesh Chandra Joshi, Mani Vetriventhan, P. Bhavani, K. B. Palanna, N. Marappa, T. E. Nagaraja, Shilpa P. Chouti, B. Nandini, R. Madhusudhana

**Affiliations:** 1 Zonal Agricultural and Horticultural Research Station, Babbur Farm, Hiriyur, Keladi Shivappa Nayaka University of Agricultural and Horticultural Sciences (KSNUAHS), Shivamogga, Karnataka, India; 2 Genebank, International Crops Research Institute for Semi-Arid Tropics (ICRISAT), Hyderabad, India; 3 Department of Genetics and Plant Breeding, Prof. Jayashankar Telangana Agricultural University (PJTAU), Rajendranagar, Hyderabad, India; 4 Indian Council of Agricultural Research (ICAR)-Vivekananda Parvatiya Krishi Anusandhan Sansthan (Vivekananda Institute of Hill Agriculture), Almora, Uttarakhand, India; 5 Department of Biotechnology, University of Agricultural Sciences, Bangalore, Karnataka, India; 6 All India Co-ordinated Research Project (AICRP) on Small Millets, University of Agricultural Sciences (UAS), Gandhi Krishi Vigyana Kendra (GKVK), Bengaluru, Karnataka, India; 7 Directorate of Research Office, University of Agricultural Sciences, Dharwad, Karnataka, India; 8 Indian Council of Agricultural Research (ICAR)- Indian Institute of Millets Research (IIMR), Hyderabad, India

**Keywords:** browntop millet, climate resilience, genetic diversity, MGIDI index, principal component analysis (PCA)

## Abstract

**Introduction:**

Browntop millet (*Urochloa ramosa* L.) is a climate-resilient crop valued for its tolerance to harsh environments and high nutritional quality. However, limited genetic improvement has restricted its wider utilization. This study aimed to assess genetic diversity and identify trait-specific donors for enhanced agronomic performance.

**Material and methods:**

A diversity panel of 121 accessions of *U. ramosa* was evaluated for key agronomic traits. Genetic parameters including variability, heritability, and genetic advance were estimated. Correlation and path coefficient analyses were performed to determine trait associations. Principal component analysis (PCA), cluster analysis, and multi-trait genotype–ideotype distance index (MGIDI) were employed to identify superior genotypes.

**Results:**

Significant genotypic variation was observed across all traits. Grain yield and fodder yield exhibited high heritability and genetic advance, indicating additive gene action. Correlation and path analysis revealed strong positive associations between yield and contributing traits. PCA showed that the first five components explained 68.66% of total variation. Cluster analysis grouped genotypes into four clusters, with Cluster IV containing superior accessions. MGIDI analysis identified 11 promising genotypes, including GPUBT 6, VBT 004, TNAU 164, IC 613548, IC 613553, TNAU 129, TNAU 110, TNAU 134, TNAU 140, HBr 2, and TNAU 150.

**Conclusion:**

The study highlights substantial genetic variability in browntop millet and identifies promising genotypes for dual-purpose improvement. These findings provide a foundation for developing high-yielding, early-maturing, and climate-resilient cultivars.

## 1 Introduction

The world population has exceeded 8 billion, with 7.8-8.8% experiencing hunger in 2024 (FAO, 2025). The effects of climate change, via biotic and abiotic stress on crops, have exacerbated the challenge of alleviating hunger. Mitigating hunger and malnutrition necessitates the cultivation of nutrient-dense and climate-resilient crops that thrives well in low input marginal agro-ecologies of South Asia and Africa. Minor millets offer high nutritional value and demonstrated adaptation to harshest growing conditions across the world (Joshi et al., 2020). The increasing acknowledgement of the nutritional and health advantages of minor millets positions them as viable choices for improving food security. However, the scarcity of research and processing methods has reduced the large scale incorporation of minor millets in the diet (Upadhyaya et al., 2016).

Browntop millet, an underappreciated orphan crop with versatile uses, requires greater attention (*Urochloa ramosa* L.). It is a tetraploid species of the Poaceae family, distinguished by basic chromosome numbers of X = 7, 8, and 9 (Nandini et al., 2025a, Nandini et al., 2025b). It originated in Southeast Asia and is now cultivated in China, Western Asia, Arabia, Africa, and Australia. *Urochloa* species are widely used as fodder and cultivated in pastures across tropical America and Southeast Asia (Valle et al., 2008) and they have also spread in several regions of the United States, particularly as a popular feed for game birds. Thus, browntop millet serves as a catch, nurse, and green manure crop (Bhavani et al., 2024; Nandini et al., 2025a). It is often grown in mixed stands, typically with other grass species and legumes, for feed purposes. Moreover, it may thrive in shaded regions and orchards (Bhat, 2018). The serrated leaf margins assist in safeguarding crop from rodent occurrence. A strong root system aids in soil conservation by preventing erosion and degradation (Anuradha et al., 2020) and acts as a supplementary measure to alleviate root-knot nematode issues in peppers and tomatoes (Ashoka and Sunitha, 2020). Browntop millet has several nutritional benefits, serving as an excellent source of health-enhancing flavonoids, tannins, resins, quinones, macronutrients, and minerals (Bhavani et al., 2024; Nandini et al., 2025a). Further, its ability to regulate blood pressure, glucose levels, and insulin efficacy (Hemamalini et al., 2021) and its gluten free nature makes it one of most suitable options for those with coeliac disease (Saleh et al., 2013). Consequently, it serves as an exceptional remedy for addressing lifestyle diseases and modern dietary preferences (Kishore et al., 2021).

Despite of its considerable nutritional benefits and adaptability to climate change, only a handful of cultivars have so far been developed and made available for cultivation by farmers. Consequently, farmers generally cultivate traditional type and low yielding heterogeneous blend of landraces with loose panicles which has rendered poor productivity levels of the crop. Therefore, cultivars adapted to diverse agro-ecologies are crucial for expanding the crop area under cultivation and germplasm collections provides the genetic base for this advancement. Approximately 2,517 accessions of browntop millet germplasm have been preserved in different national and international gene banks worldwide (Nandini et al., 2025a). The all India coordinated research project on small millets (AICRP-SM) is a national active germplasm site of small millets and maintain 123 *Urochloa ramosa* (L.) germplasm accessions (Bhavani et al., 2024). However, studies on the phenotypic characterization of browntop millet germplasm are notably limited, only the phenotypic characterization of 25 accessions has been carried out (Rahul et al., 2024). This critical impediment of lack of detailed characterization information of accessions is hampering the large scale utilization of these genetic resources in crop breeding.

To tailor an improved and widely adaptable cultivar in browntop millet, the primary breeding objectives include high grain yield, high tillering, compact panicles, enhanced culm strength, ease of dehulling, and improved grain size. A rigorous evaluation of diverse germplasm collections of minor millets, including little millet (Vetriventhan et al., 2021), proso millet (Vetriventhan and Upadhyaya, 2018), barnyard millet (Sood et al., 2015) and kodo millet (Vetriventhan and Upadhyaya, 2019) has revealed trait-specific accessions suitable for cultivar development. Consequently, characterization information generated and the identification of trait-specific donors are crucial for this minor crop and can fill existing gaps.

This is the first study to evaluate genetic variation and identify trait-specific donors for improved agronomic traits within a comprehensive diversity panel consisting of 121 *U. ramosa* accessions. Majorly Multi-Trait Genotype–Ideotype Distance Index (MGIDI) enables the selection of best-performing genotypes for multiple traits through optimized ranking based on proximity to the genotypes to the ideal genotype (Olivoto and Nardino, 2021). This overcomes the limitation of existing single trait based selection. This provides a robust framework and strengthens the browntop millet breeding program for development of high yielding and widely adaptable browntop millet cultivars. Against this backdrop, in the present study includes characterization and assessment of genetic variation for agronomic traits among 121 diverse browntop millet germplasm, understanding the trait associations among yield and related traits and finally identification of trait-specific donors using multi-trait selection approaches to strengthen breeding programs for this minor yet sturdy millet.

## 2 Materials and methods

### 2.1 Plant material

**Figure 1:**
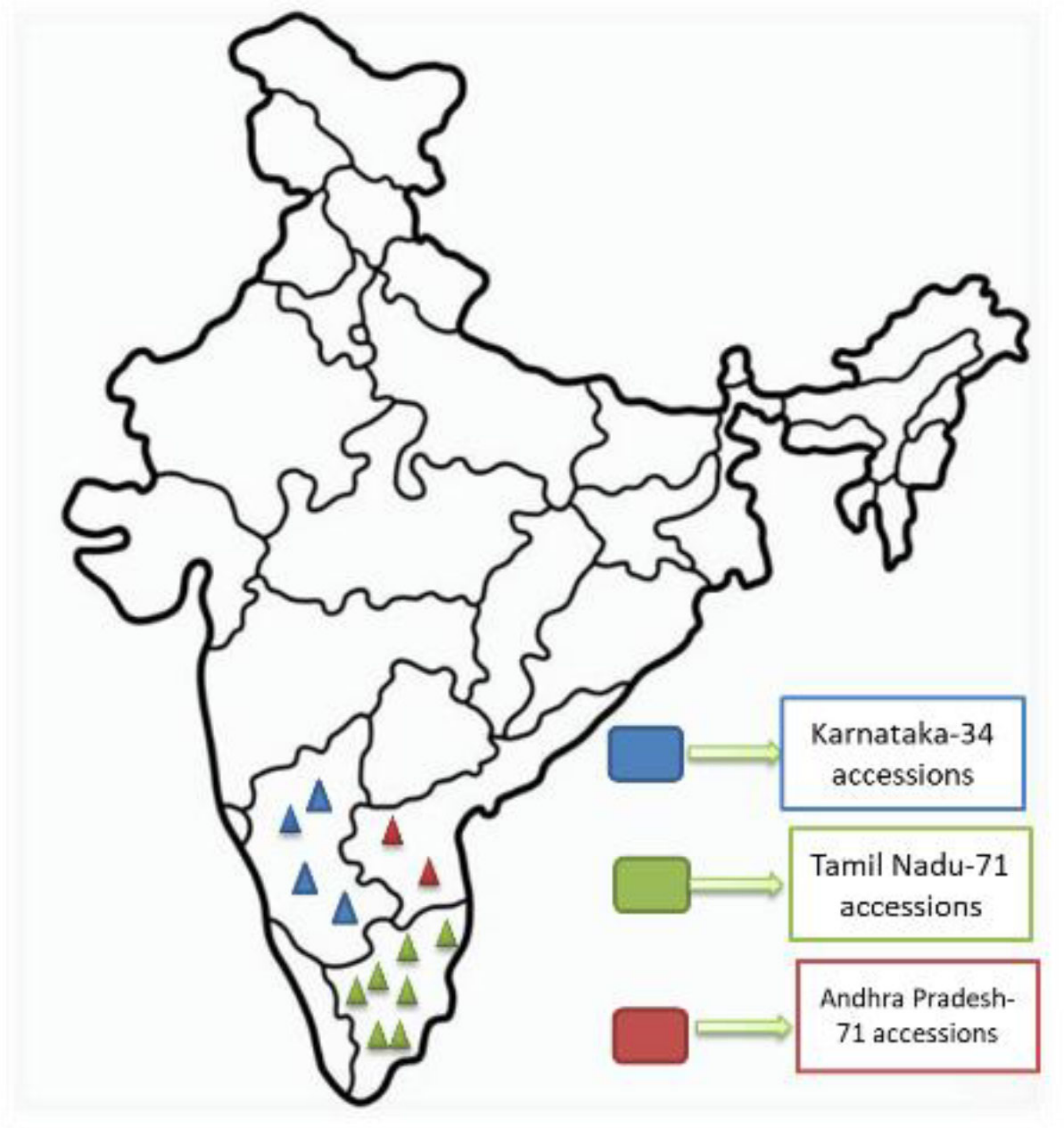
Map depicting the geographic location of browntop millet germplasm collection used in the present study The map represents the collection sites of browntop millet germplasm from different states of India. The three different color represents the three different states form where germplasm has been collected as presented. This distribution highlights the diversity of accessions evaluated for agronomic traits in the present study.

The present study utilized 121 genotypes of browntop millet, comprising 111 germplasm accessions, 8 advanced breeding lines, and 2 released cultivars. The accessions were obtained from the National Active Germplasm Site (NAGS), Project Coordinating Unit, AICRP on Small Millets, University of Agricultural Sciences, GKVK, Bengaluru, and represent the entire germplasm collection of browntop millet in India. These accessions were majorly collected from states of Karnataka, Tamil Nadu and Andhra Pradesh of India (**Figure 1****).** This crop, which originated and is primarily cultivated in India, has led to the collection and storage of numerous accessions within the country, representing the complete diversity of *Urochloa ramosa*. This is the first study to characterize all existing germplasm of this crop. **Supplementary Table 1** presents the accessions along with their respective collection sites.

### 2.2 Experimental details

Field experiments were conducted on clay loam black soil over two consecutive rainy seasons (2023 and 2024) at the Zonal Agricultural Horticultural Research Station, Babbur Farm, Hiriyur, situated in the Central Dry Zone of Karnataka, India. The experimental site is located at 13°57’ North latitude and 75°38’ East longitude, with an altitude of 606 meters above mean sea level (MSL). The experiment employed an alpha lattice design featuring two replications. The accessions were established during the second week of July in both the years. Each accession was planted in a single row measuring 4 m in length, with a spacing of 45 cm between rows and approximately 10 cm between plants, yielding approximately 40 plants per accession. Fertilizers were applied at the rate of 30 kg N_2_ ha-1, 30 kg P_2_O_5_ ha-1, and 20 kg K_2_O ha-1 as a basal dose, with an additional 30 kg N_2_ ha-1 applied as topdressing. Hand weeding and thinning was performed 21 days post-sowing. Irrigation and plant protection measures were implemented as necessary. In 2023, rainfall during the cropping periods measured 233.2 mm, while in 2024, it was 636.4 mm. In 2023, the maximum temperature varied between 28.9 °C and 33.1 °C, while in 2024, it ranged from 29.3 °C to 32.4 °C. The minimum temperature in 2023 was between 18.4 °C and 21.2 °C, compared to 17.5 °C to 21.7 °C in 2024 during the crop growth period.

### 2.3 Data collection

Qualitative traits were assessed based on the descriptors for little millet (*Panicum sumatrense*) as outlined by the IBPGR (1986). Descriptors for *Urochloa ramosa* have not been published; therefore, those for little millet were utilized, as this crop is a close relative of little millet. Qualitative traits data were recorded on a plot basis through visual observations. This includes plant pigmentation at the node (pigmented and green), leaf sheath (pigmented and green), and auricle (pigmented and green), as well as inflorescence shape (compact and open) and compactness (compact, intermediate, and loose) and panicle pubescence (absence, intermediate and present). Data were collected on 12 quantitative traits for the years 2023 and 2024. Data for days to flowering and days to maturity were recorded on a plot basis. In contrast, traits including plant height (cm), number of panicles per plant, number of basal tillers, flag leaf blade length (cm), flag leaf blade width (cm), panicle width (cm), panicle length (cm), 1000 seed weight (g), grain yield per plant (g), and fodder yield per plant (g) were recorded on a five-plant basis, with average values utilized for analysis.

### 2.4 Statistical analysis

The data collected for 12 quantitative traits during both years were analyzed individually and pooled over two seasons using the residual maximum likelihood (REML) approach in GenStat 24^th^ edition, considering genotypes as random and seasons as fixed (https://vsni.co.uk/software/genstat/). The significance of seasons was tested using Wald’s statistics. The genetic parameters such as heritability (h^2^b), genotypic and phenotypic coefficients of variation (GCV and PCV), genetic advance, and genetic advance as a percentage of the mean were calculated based on the variance components of genotype (σ^2^g), genotype × season (σ^2^g*s), and error variance. The h^2^b was categorized as low (<0.30), medium (0.30–0.60), and high (>0.60); while GCV and PCV values were categorized as low (<10%), moderate (10–20%) and high (>20%) (Deshmukh et al., 1986). The genetic advance as % mean was categorized low (0-10%), medium (10-20%) and high (>20%) (Johnson et al., 1955).The best linear unbiased predictors (BLUPS) were estimated for various agronomic traits considered for individual and pooled seasons and were used for further analysis. The distribution of individual seasons was plotted using violin-boxplot plots using the ggplot2 package (version 3.5.1) of R software. Correlation coefficients were estimated using the metan package (version: 1.18.0), and principal component analysis was performed using the factoextra package (version: 1.0.7) in R software and traits with higher absolute loading values (≥ 0.50) were considered to have strong associations with the respective principal components, while trait contributions (%) were used to interpret the relative importance of traits within each component. components with eigenvalues greater than 1.0 were retained according to the Kaiser criterion (Kaiser, 1960). Hierarchical clustering was performed based on the Euclidean distance and Ward’s method using the ggplot2 package (version 3.5.1). The trait specific accessions for days to maturity, grain yield per plant (g), fodder yield per plant (g), and 1000 seed weight (g) were identified through comparison with trial mean of respective traits using LSD (5%).

The Multi-Trait Genotype–Ideotype Distance Index (MGIDI) was computed to identify superior genotypes based on multiple traits simultaneously. The pooled blup means of genotypes were used for selection of superior genotypes using MGIDI. Traits included in the MGIDI analysis were selected based on their significant correlation with grain yield. These traits included plant height, number of tillers per plant, panicles per plant, panicle length, and thousand seed weight. Trait desirability was defined according to their breeding importance, where lower values were considered desirable for plant height to reduce lodging risk, while higher values were considered desirable for the number of tillers per plant, panicles per plant, panicle length, and thousand seed weight due to their positive association with grain yield. The ideotype was defined based on these desired trait directions. The MGIDI index was computed using the metan package in R software (Olivoto & Nardino, 2021), which uses factor analysis to group correlated traits and estimates the Euclidean distance of each genotype from the ideotype. Genotypes with lower MGIDI values were considered closer to the ideal genotype and therefore more desirable.

## 3 Results

### 3.1 Variance components and heritability

The observed variability among the genotypes evaluated for various morphological traits was evidenced by highly significant genotypic variance components across both the years i.e., 2023 and 2024, as well as in the pooled data (**Table 1****).** Furthermore, the highly significant Wald statistic underscores the considerable influence of seasonal variation on trait performance of the genotypes. However, the genotype × season interaction did not significantly affect most traits, with the exception of grain and fodder yields per plant. Among the various traits, high heritability was noted for days to flowering, days to maturity, plant height, 1000 seed weight, grain yield, and fodder yield during both years and in the pooled data. Moderate heritability was noted for the trait of panicle number per plant in 2023 and 2024, while high heritability was observed under the pooled analysis. Likewise, the number of basal tillers per plant exhibited moderate heritability in the year 2024, but high heritability in 2023 and under pooled analysis. Similarly, the traits flag leaf blade length, panicle length and panicle width showed moderate heritability in 2023, but high in heritability in 2024 and also in the pooled analysis.

**Table 1:** Genotypic (*σ*^2g^), genotype × season interaction (*σ*^2gs^) variance components, and broad-sense heritability (*h*^2b^) estimates for agronomic traits in browntop millet germplasm.

Traits	Individual season				Combined over two seasons			
	2023		2024		*σ* ^2g^	*σ* ^2gs^	Wald statistic	*h* ^2b^
	*σ* ^2g^	*h* ^2b^	*σ* ^2g^	*h* ^2b^				
Days to Flowering	7.23^***^	0.92	8.24^***^	0.96	7.83^***^	0.00 ^NS^	2041.88^***^	0.98
Days to Maturity	6.86^***^	0.92	7.78^***^	0.95	7.30^***^	0.00 ^NS^	1220.77^***^	0.98
Plant Height (cm)	67.92^***^	0.66	57.82^***^	0.65	66.40^***^	0.00 ^NS^	365.98^***^	0.88
Number of panicles per plant	0.39^***^	0.61	0.43^***^	0.53	0.40^***^	0.028 ^NS^	70.64^***^	0.81
Number of Basal Tillers	0.46^***^	0.48	0.64^***^	0.55	0.49^***^	0.080^NS^	135.72^***^	0.74
Flag leaf blade length (cm)	1.19^***^	0.55	0.77^**^	0.28	0.90^***^	0.00^NS^	24.17^***^	0.66
Flag leaf blade width (cm)	0.01^***^	0.43	0.03^***^	0.70	0.02^***^	0.00 ^NS^	5.59^**^	0.87
Panicle width (cm)	0.01^***^	0.34	0.02^***^	0.67	0.02^***^	0.002 ^NS^	16.93^***^	0.71
Panicle length (cm)	0.93^***^	0.34	1.27^***^	0.62	1.18^***^	0.00 ^NS^	9.58^**^	0.78
1000 Seed weight (g)	0.04^***^	0.75	0.03^***^	0.69	0.04^***^	0.002 ^NS^	247.01^***^	0.90
Grain Yield per plant (g)	10.39^***^	0.80	17.67^***^	0.85	10.71^***^	3.32^***^	48.07^***^	0.82
Fodder yield per plant (g)	569.70^***^	0.93	331.78^***^	0.88	424.52^**^	42.08^***^	0.03 ^NS^	0.93

*, ** and *** indicates level of significance at 5%, 1% and 0.1% respectively and NS indicates non-significance.

### 3.2 Mean, range and variability parameters

**Figure 2:**
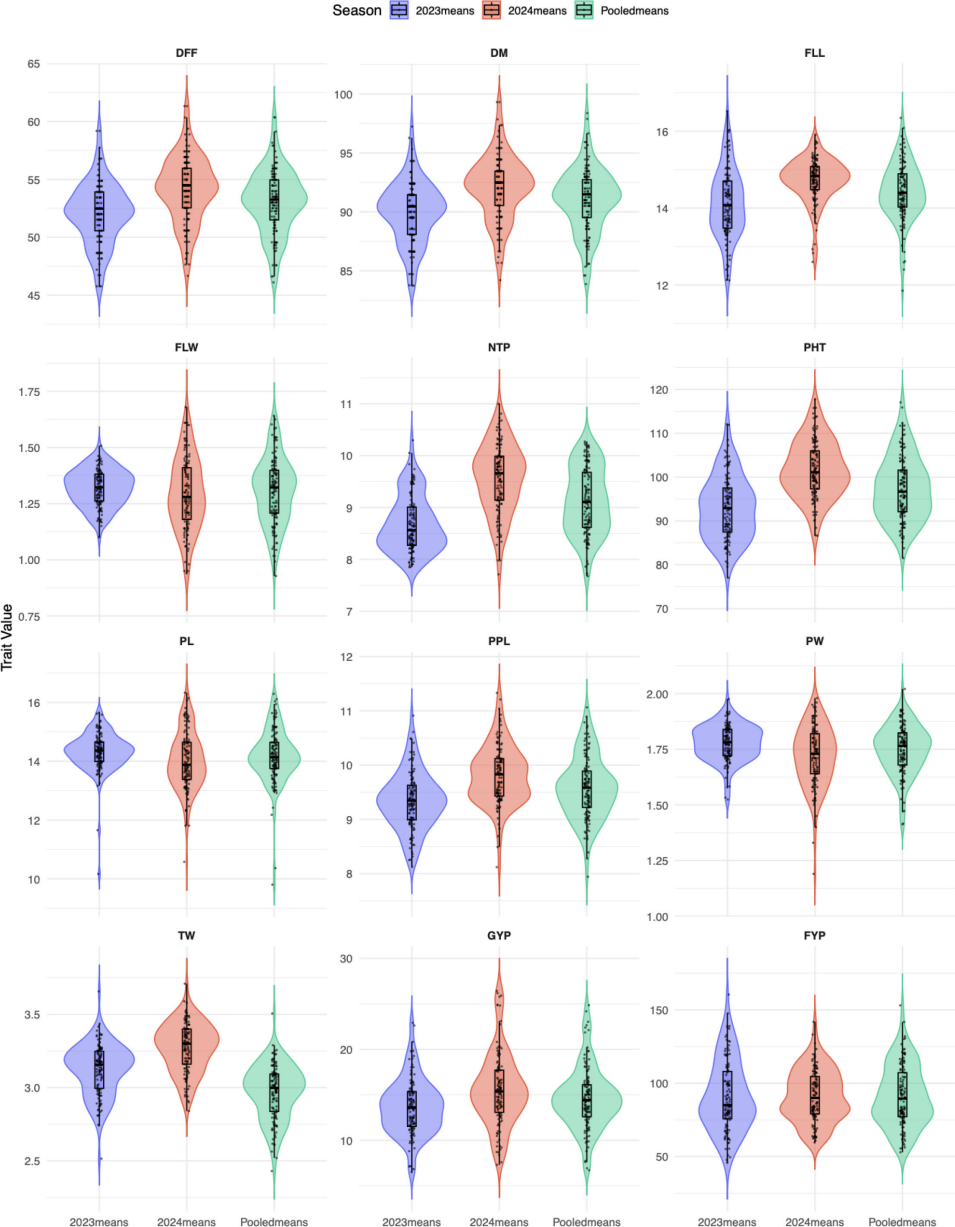
Violin-box plot representing distribution of various agro-morphological traits of browntop millet germplasm evaluated across two seasons (2023–2024). The violin plots illustrate the distribution of each trait, while the box plots represent the median (central line), interquartile range (box), and minimum–maximum values (whiskers). The analysis summarizes phenotypic variability observed across seasons for key growth, yield, and yield-related traits. DFF = days to flowering; DM = days to maturity; PHT, plant height (cm); FLL, flag leaf length (cm); FLW, flag leaf width (cm); NTP, number of basal tillers per plant; PPL, panicles per plant; PL, panicle length (cm); PW, panicle width (cm); GYP, grain yield per plant (g); FYP, fodder yield per plant (g); TW, thousand seed weight (g).

The mean and range derived from the estimated Blups were tabulated as **Table 2**, while variability parameters determined from the variance components were presented in **Table 3**. The performance of genotypes across various traits and their distribution over both years, as well as when pooled, are illustrated using a violin-box plot (**Figure 2****).** Significant variation was observed in most of the evaluated traits. The variation in grain yield was notably high in both seasons, being threefold higher with a mean of 13.62 g (ranging from 6.50 to 22.91 g) in 2023, 15.56 g (ranging from 7.30 to 26.43 g) in 2024, and 14.49 g (ranging from 6.70 to 24.85 g) under pooled analysis. The mean and range of other traits, along with the coefficient of variation and least significant differences, are shown in **Table 2**. A significant genetic advance as a percentage of the mean was seen for both grain and fodder yield per plant in pooled data as well as in individual years. A moderate genetic advance as a percentage of the mean was observed for traits including days to flowering, plant height (cm), number of panicles per plant, and number of basal tillers per plant, while a low genetic advance as a proportion of the mean was recorded for days to maturity in both individual seasons and when aggregated. In 2023, a low genetic advance as a percentage of the mean was noted for panicle length and breadth, whereas a considerable genetic advance was observed in 2024 and when data were pooled. A moderate genetic advance as a percentage of the mean was noted for flag blade length in 2023 and in the pooled data, whereas it was low in 2024. Furthermore, it was moderate in 2023 and high in 2024, and aggregated for flag leaf blade width. A substantial genetic advance as a percentage of the mean was observed for 1000 seed weight in 2023 and pooled analysis, but it was low in 2024.

**Table 2:** Range and mean of agronomic traits in brown top millet germplasm.

Traits	Mean			Range			LSD (5%)			CV %		
	2023	2024	Pooled	2023	2024	Pooled	2023	2024	Pooled	2023	2024	Pooled
Days to Flowering	52	54	53	45-59	47-61	46-60	1.56	1.22	1.40	1.52	1.13	1.33
Days to Maturity	90	92	91	84-97	84-99	84-98	1.53	1.28	1.41	0.86	0.70	0.78
Plant Height (cm)	92.97	101.8	97.38	77-112	86.65-117.79	81.55-117.06	11.64	10.98	11.79	6.32	5.45	6.11
Number of panicles per plant	9.34	9.81	9.57	8-11	8.12-11.33	8-11.06	1.0	1.23	1.16	5.39	6.33	6.12
Number of basal tillers	8.68	9.57	9.12	7.85-10.30	7.71-11	7.68-10.27	1.4	1.44	1.46	8.16	7.63	8.06
Flag leaf blade length (cm)	14.13	14.73	14.43	12.12-16.52	12.60-15.91	11.85-16.34	1.95	2.76	2.69	6.98	9.48	9.40
Flag leaf blade width (cm)	1.32	1.29	1.31	1.10-1.51	0.94-1.68	0.93-1.64	0.24	0.23	0.23	9.31	8.91	9.02
Panicle width (cm)	1.78	1.72	1.75	1.52-1.98	1.19-1.98	1.41-2.02	0.32	0.22	0.28	9.12	6.47	8.13
Panicle length (cm)	9.34	14.01	14.17	8.12-10.91	10.58-16.33	9.80-16.29	1.00	1.74	2.28	5.39	6.27	8.12
1000 Seed weight (g)	3.12	3.27	3.20	2.51-3.66	2.84-3.71	2.43-3.51	0.22	0.25	0.21	3.48	3.79	3.33
Grain yield per plant (g)	13.62	15.56	14.59	6.50-22.91	7.30-26.43	6.70-24.85	3.20	3.46	3.33	11.86	11.23	11.51
Fodder yield per plant (g)	91.01	91.20	91.11	45.76-160.41	59.59-141.79	52.94-153.03	13.56	13.61	14.13	7.53	7.54	7.84

**Table 3:** Genetic variability parameters of agronomic traits in browntop millet germplasm.

Traits	GCV (%)			PCV (%)			Genetic advance (%)			GA as % mean		
	2023	2024	Pooled	2023	2024	Pooled	2023	2024	Pooled	2023	2024	Pooled
Days to Flowering	5.16	5.3	5.27	5.38	5.42	5.31	5.31	5.78	5.72	10.21	10.67	10.77
Days to Maturity	2.91	3.03	2.98	3.03	3.11	3.00	5.18	5.60	5.54	5.75	6.08	6.08
Plant Height (cm)	8.86	7.47	8.37	10.89	9.25	8.91	13.82	12.66	15.77	14.87	12.43	16.19
Number of panicles per plant	6.70	6.71	6.62	8.60	9.22	7.38	1.00	0.99	1.17	10.76	10.04	12.25
Number of Basal Tillers	7.84	8.38	7.67	11.32	11.33	8.94	0.97	1.22	1.24	11.18	12.78	13.55
Flag leaf blade length (cm)	7.72	5.97	6.57	10.40	11.20	8.08	1.67	0.96	1.59	11.79	6.55	11.01
Flag leaf blade width (cm)	8.13	13.62	11.93	12.36	16.27	12.75	0.15	0.30	0.30	11.02	23.47	22.98
Panicle width (cm)	6.54	9.15	7.17	11.22	11.21	8.52	0.14	0.26	0.22	7.84	15.38	12.42
Panicle length (cm)	6.74	8.04	7.66	11.60	10.20	8.67	1.15	1.83	1.98	8.06	13.07	13.95
1000 Seed weight (g)	6.03	5.66	6.46	6.96	6.81	6.82	0.34	0.32	0.37	10.76	9.68	12.59
Grain Yield per plant (g)	23.66	27.01	22.43	26.47	29.25	24.78	5.94	8.00	6.10	43.58	51.39	41.82
Fodder yield per plant (g)	26.84	19.97	22.62	27.88	21.35	23.50	48.45	35.11	40.85	53.24	38.49	44.84

### 3.3 Variability for qualitative traits

**Figure 3:**
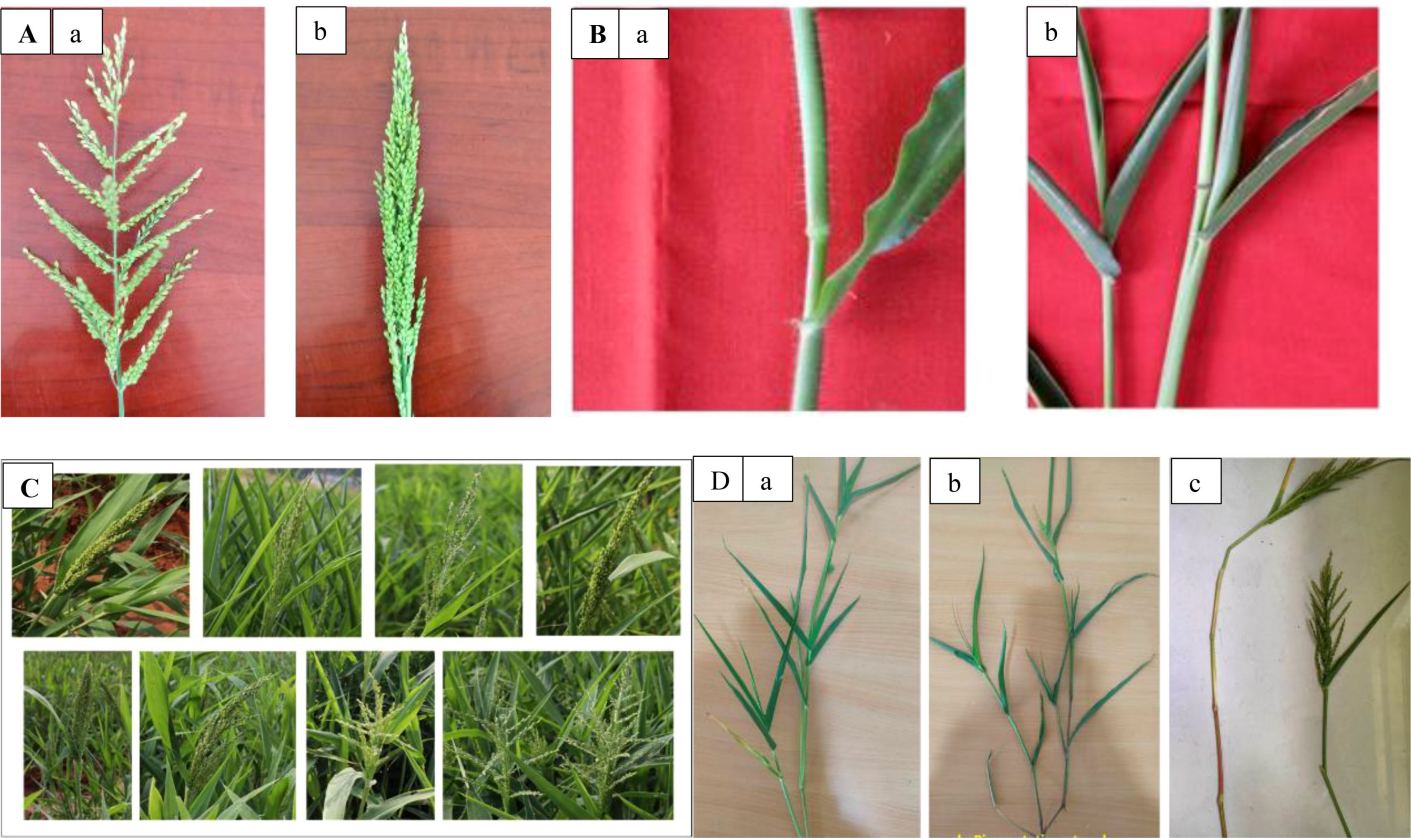
Variations observed for qualitative traits in browntop millet (*Urochloa ramosa* L.) germplasm accessions. **(A)** Types of panicles (a) Open-Chaduru (b) Compact-Dundu. **(B)** Variations fore nodal pigmentation (a) Non pigmented at node (b) Pigmentation at node. **(C)** Variations for panicle compactness. **(D)** Variations for leaf sheath and nodal pigmentation (a) Non pigmented leaf sheath (b) pigmentation at node (c) pigmentation at leaf sheath. **(A–C)** Reproduced from Nandini et al. (2025a). Copyright Wiley‐VCH GmbH, reproduced under license by John Wiley and Sons.

The distribution of phenotypic categories for the six qualitative traits exhibited considerable variation among the Browntop millet germplasm (**Table 4**). A large proportion of genotypes were classified as green non pigmented types in nodes (86.78%), auricles (89.26%), and leaf sheaths (90.90%), whereas pigmented types were relatively less frequent. The predominance of non-pigmented types suggests that these traits are relatively conserved within the evaluated germplasm, while the presence of pigmented variants indicates existing genetic variability that could be useful for morphological identification and germplasm characterization. With respect to inflorescence morphology, genotypes were grouped into compact and open types, with compact forms being more frequent (54.55%) than open forms (45.45%), indicating moderate variation in panicle architecture. In terms of inflorescence compactness, the distribution of genotypes into compact (47.93%), intermediate (23.97%), and loose (28.09%) categories suggests substantial diversity for this trait, which may influence grain filling and yield performance under different environmental conditions. For panicle pubescence, most genotypes exhibited pubescent panicles (45.46%), followed by intermediate (31.40%) and non-pubescent types (23.14%), reflecting variability that may be associated with adaptation to environmental stresses and pest tolerance. The Shannon–Wiener diversity index (H′) further confirmed the presence of variability among the qualitative traits, with values ranging from 0.30 for leaf sheath coloration to 1.05 for inflorescence compactness and an overall mean of 0.58, indicating moderate phenotypic diversity within the germplasm. Such variation in qualitative traits provides useful descriptors for germplasm characterization and may support the identification of diverse parental lines for breeding programs. The observed variations for these qualitative traits are illustrated in **Figure 3**.

**Table 4:** Frequency of phenotypic classes of each qualitative trait in the browntop millet germplasm collection.

Sl. no.	Trait	Classification	Number of accessions	Frequency (%)	Shannon-Weiner diversity (H`)
1	Leaf sheath pigmentation	Pigmented	11	9.09	0.30
		Non-pigmented	110	90.90	
2	Auricle pigmentation	Pigmented	13	10.74	0.34
		Non-pigmented	108	89.26	
3	Nodal pigmentation	Pigmented	16	13.22	0.39
		Non-pigmented	105	86.78	
4	Inflorescence shape	Compact (Dundu)	66	54.55	0.69
		Open (Chaduru)	55	45.45	
5	Inflorescence compactness	Compact	58	47.93	1.05
		Intermediate	29	23.97	
		Loose	34	28.09	
6	Panicle pubescence	Absence	28	23.14	0.68
		Intermediate	38	31.40	
		Present	55	45.46	
	Mean across traits				0.58

### 3.4 Trait association studies

**Figure 4:**
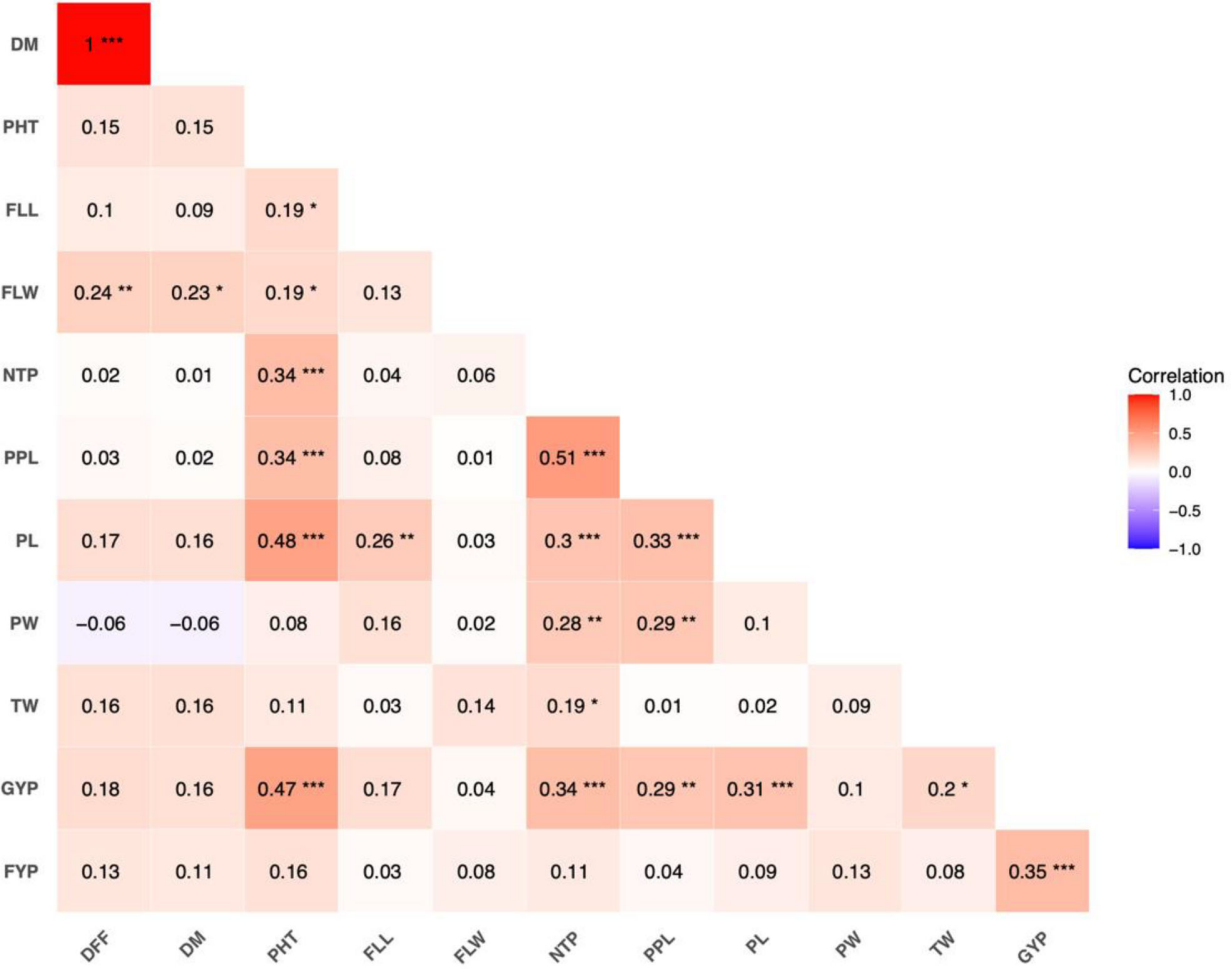
Correlation analysis between grain yield and agro-morphological traits in 121 browntop millet (*Urochloa ramosa* L.) germplasm accessions. The correlation matrix illustrates the direction and strength of associations among grain yield per plant and related morphological traits. Correlation coefficients are represented using a color gradient scale shown on the right side of the figure, where color intensity reflects the magnitude of correlation. Asterisks indicate statistical significance (*p* < 0.05, p < 0.01, *p* < 0.001). DFF, days to flowering; DM, days to maturity; PHT, plant height (cm); FLL, flag leaf length (cm); FLW, flag leaf width (cm); NTP, number of basal tillers per plant; PPL, panicles per plant; PL, panicle length (cm); PW, panicle width (cm); GYP, grain yield per plant (g); FYP, fodder yield per plant (g); TW, thousand seed weight (g).

The correlation analysis has unveiled several existing associations among evaluated traits (**Figure 4****).** Grain yield was positively correlated with plant height (0.47***), number of tillers per plant (0.34***), panicles per plant (0.29**), panicle length (0.31*), thousand seed weight (0.2*) and fodder yield per plant (0.35***). Non-significant positive association was observed for days to flowering, days to maturity, flag leaf length, flag leaf width and panicle width. In addition to grain yield per plant, plant height showed a positive association with flag leaf length (0.19**), number of tillers per plant (0.34**), panicles per plant (0.34**), and panicle length (0.48***). Moreover, the number of tillers per plant was positively correlated with panicles per plant (0.51***), panicle length (0.3***), and panicle width (0.28**). Panicles per plant also showed a noteworthy positive association between panicle length (0.33***) and panicle width (0.29**). To further understand the cause of the complex associations between the traits, path analysis was carried out, which revealed direct and indirect effects. Prior to the path analysis, the inter-trait correlations were examined and no severe multicollinearity (r > 0.80) was observed among independent traits. The outcome of path analysis in the form of contributions of various traits towards correlation with grain yield through direct and indirect effects is tabulated in **Supplementary Table 2**. It was identified that the positive association between the traits of plant height, number of tillers per plant, panicle length, panicles per plant and fodder yield per plant and grain yield was due to direct effects only.

### 3.5 Principal component analysis

**Figure 5:**
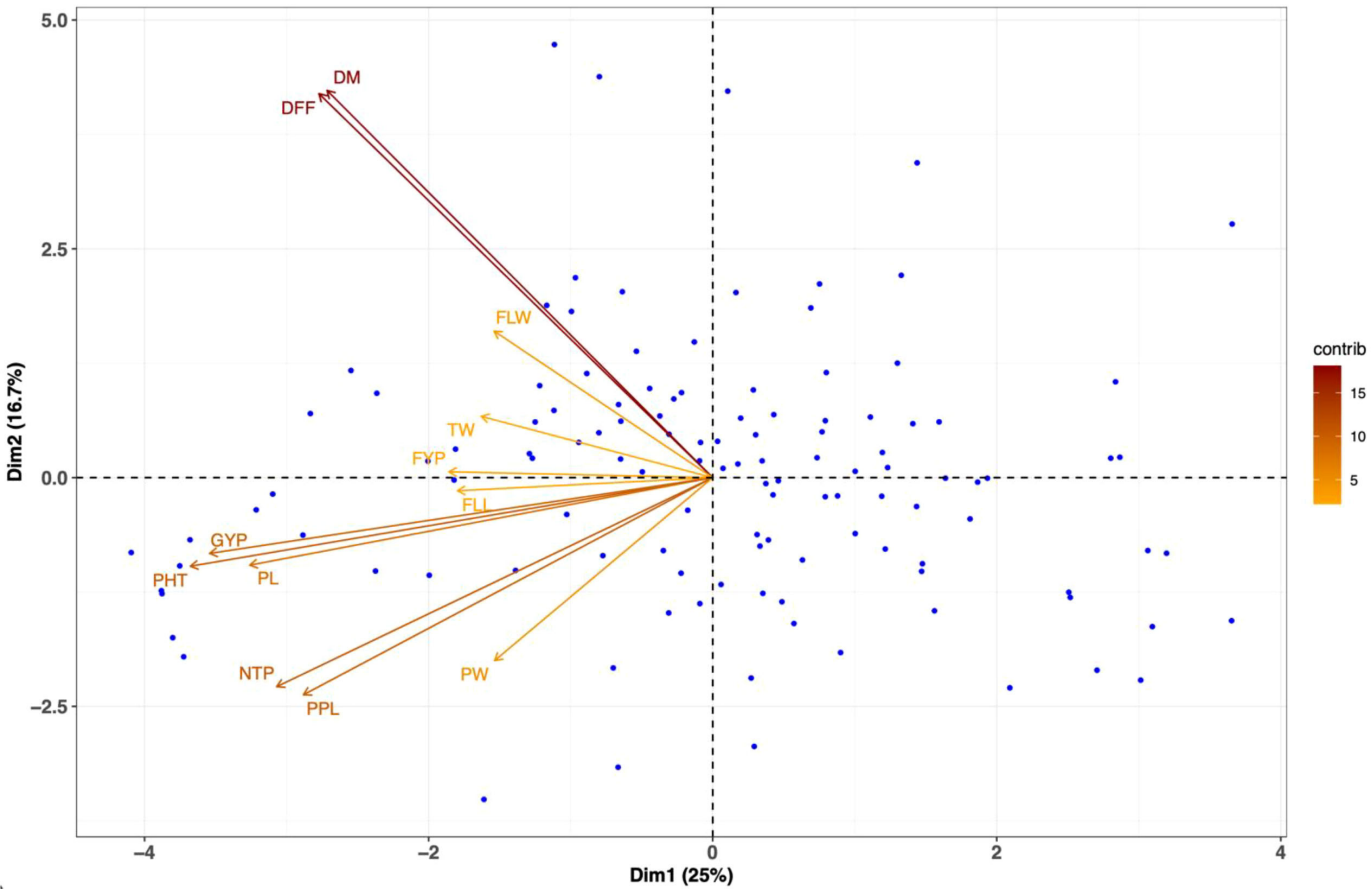
Principal component analysis (PCA) biplot representing the distribution of genotypes and agro-morphological traits in 121 browntop millet (*Urochloa ramosa* L.) germplasm accessions. The biplot depicts the relationship among genotypes and traits evaluated based on the first two principal components (PC1 and PC2), which explain the maximum proportion of total phenotypic variation. Genotypes are represented as points, and trait vectors indicate the direction and magnitude of their contribution to variability. The angle between vectors reflects the correlation among traits, with acute angles indicating positive associations and obtuse angles indicating negative associations. DFF, days to flowering; DM, days to maturity; PHT, plant height (cm); FLL, flag leaf length (cm); FLW, flag leaf width (cm); NTP, number of basal tillers per plant; PPL, panicles per plant; PL, panicle length (cm); PW, panicle width (cm); GYP, grain yield per plant (g); FYP, fodder yield per plant (g); TW, thousand seed weight (g).

In the principal component analysis, the first five principal components, each with an eigenvalue greater than 1 and accounting for more than 5% of the variance, were selected. Collectively, these five principal components explained 68.66% of the total variability in the evaluated germplasm (**Table 5****).** The first principal component (PC1) was highly loaded by the parameters of plant height, grain yield per plant, panicle length, number of basal tillers per plant, days to maturity, and days to flowering. Additionally, the traits of days to 50% flowering and days to maturity were majorly determine the variation in the second principal component (PC2). The third principal component (PC3) was mainly characterized by the traits of thousand seed weight, fodder yield per plant, and panicle length. In the fourth principal component (PC4), the fodder yield per plant, panicle width, and grain yield per plant were the major contributors to diversity. Finally, the fifth principal component (PC5) was significantly influenced by flag leaf length and number of panicles per plant. The contribution of the traits towards the diversity can be observed from the principal component biplot for the traits evaluated traits (**Figure 5**).

**Table 5:** Principal component analysis of grain yield and various other agro-morphological characteristics of Browntop millet germplasm.

Principal component	PC1	PC2	PC3	PC4	PC5
Eigen value	3.00	2.01	1.14	1.05	1.04
Variance %	24.98	16.72	9.52	8.75	8.69
Cumulative variance %	24.98	41.70	51.22	59.97	68.66
Individual Trait contributions (%) towards PCs					
Traits	**PC1**	**PC2**	**PC3**	**PC4**	**PC5**
Days to flowering	**9.20**	**31.48**	0.35	0.83	4.17
Days to maturity	**8.82**	**32.01**	0.39	0.90	4.30
Plant height	**16.19**	1.68	2.89	4.49	0.48
Flag leaf length	3.86	0.04	**13.56**	0.96	**44.85**
Flag leaf width	2.83	4.58	0.92	9.13	**19.47**
Number of basal tillers per plant	**11.28**	**9.33**	2.03	5.44	7.40
Panicles per plant	**9.94**	**10.07**	1.11	6.05	**11.91**
Panicle length	**12.72**	1.64	**18.31**	2.15	0.01
Panicle width	2.82	**7.15**	4.93	**22.15**	2.72
Grain yield per plant	**15.02**	1.23	3.07	**16.92**	0.04
Thousand seed weight	3.18	0.80	**29.93**	4.96	1.30
Fodder yield per plant	4.13	0.01	**22.52**	**26.01**	3.36

Bold values indicate traits with a significant contribution to the variance explained by the corresponding principal component.

### 3.6 Cluster analysis

**Figure 6:**
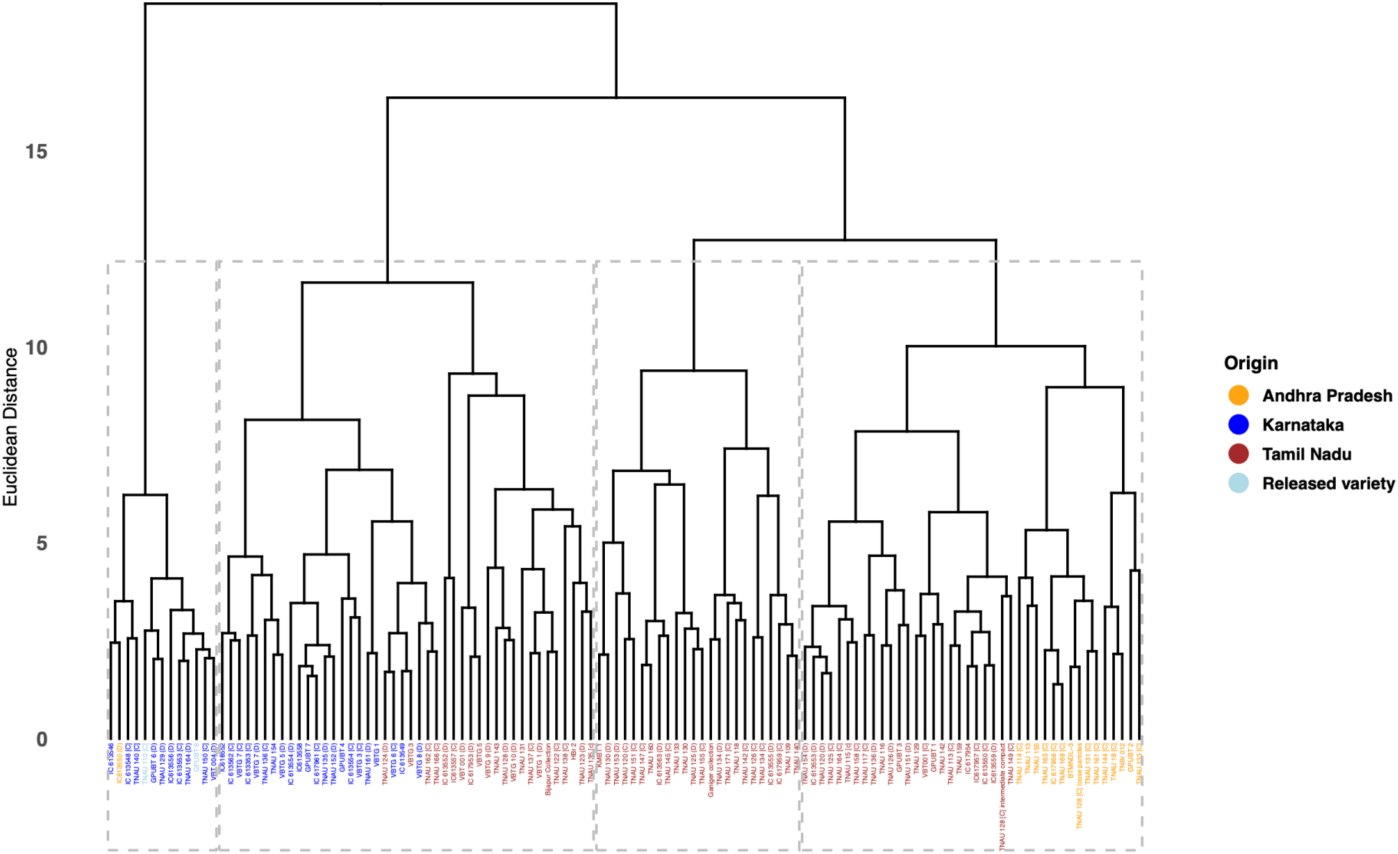
Hierarchical clustering of 121 browntop millet (*Urochloa ramosa* L.) germplasm accessions based on agro-morphological traits. The dendrogram was based on Euclidean distance and Ward’s linkage method to classify genotypes based on phenotypic similarity. The genetic divergence among accessions evaluated for yield and associated morphological traits can be observed from clusters. Genotypes grouped within the same cluster exhibit greater similarity, while those in distinct clusters indicate higher divergence. Genotype labels are color-coded based on their geographic origin: orange represents accessions from Andhra Pradesh, blue represents Karnataka, red represents Tamil Nadu, and light blue represents released varieties.

The application of Euclidean distance-based Ward’s method for hierarchical clustering has resulted in the classification of browntop millet genotypes into four distinct clusters (**Figure 6****).** Among these, Cluster 1 contained the highest number of accessions, totaling 44, while Cluster 4 comprised the lowest accessions, with only 13 genotypes. The cluster mean values of various evaluated traits are presented in **Table 6**. Clusters 2 and 3 consisted of 40 and 24 genotypes, respectively. The early flowering accessions were grouped under cluster 3 with days to flowering mean of 49 and cluster mean days to maturity of 87. Regarding plant height, Cluster 4 included the tallest genotypes, with a cluster mean of 107.83 cm, whereas the mean plant height was relatively consistent across the other three clusters. Cluster 2 demonstrated a higher fodder yield per plant, with a mean of 100.27 g. The accessions in Cluster IV genotypes were distinguished not merely by higher grain yield, but by a favorable combination of agronomic traits including taller plant stature (107.83 cm), higher fodder yield per plant (101.34 g), and higher thousand-seed weight (3.10 g). This trait combination suggests suitability of accessions under cluster 4 for dual-purpose breeding programs targeting both grain productivity and biomass yield under dryland conditions.

**Table 6:** Cluster means of grain yield and various other agro-morphological characteristics of browntop millet germplasm.

Traits	Cluster 1	Cluster 2	Cluster 3	Cluster4
Days to flowering	55	53	49	54
Days to maturity	923	91	87	92
Plant height (cm)	96.78	94.93	96.92	107.83
Flag leaf length (cm)	14.51	14.13	14.47	14.97
Flag leaf width (cm)	1.38	1.21	1.3	1.37
Number of basal tillers per plant	9.1	8.98	8.98	9.89
Panicles per plant	9.49	9.58	9.41	10.14
Panicle length (cm)	13.95	14.06	14.08	15.39
Panicle width (cm)	1.71	1.77	1.75	1.83
Grain yield per plant (g)	13.99	14.52	12.32	21.06
Fodder yield per plant (g)	86.67	100.27	78.43	101.34
Thousand seed weight (g)	3.04	2.86	2.95	3.10

The selection of contrasting and diverse genotypes from superior clusters can play a key role in hybridization programs.

### 3.7 Multi-trait genotype ideotype distance index

**Figure 7:**
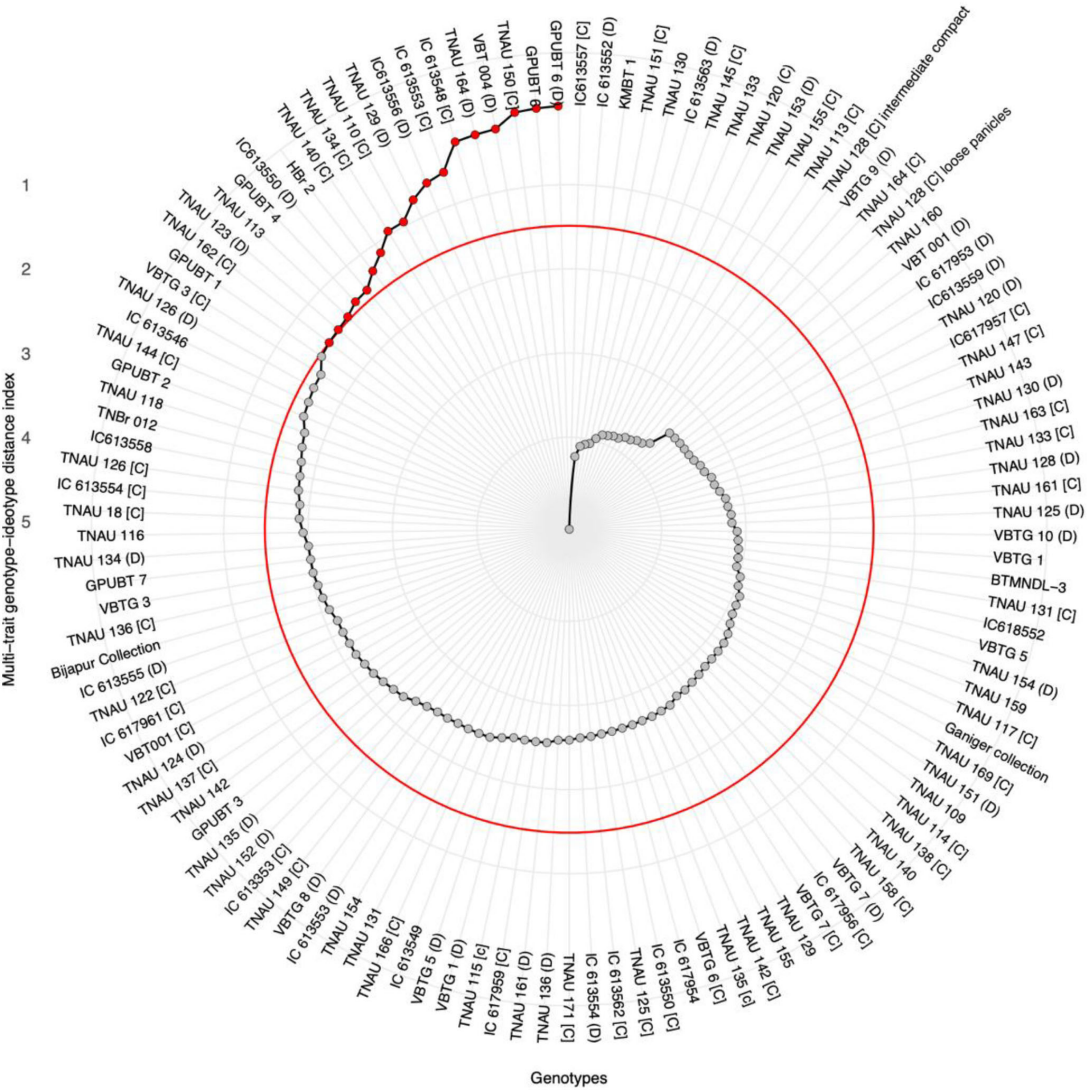
Multi-trait genotype–ideotype distance index (MGIDI) plot for selection of superior browntop millet (*Urochloa ramosa* L.) germplasm accessions based on grain yield and associated agro-morphological traits. The MGIDI index ranks genotypes according to their proximity to an ideal genotype (ideotype) with desirable trait combinations. Genotypes are arranged radially according to their MGIDI values, with lower values indicating greater proximity to the ideotype and superior multi-trait performance. Each point represents an individual genotype. The predefined selection intensity was represented in the form of red horizontal threshold line (circular boundary), and genotypes highlighted in red fall represents selected superior group. Grey points represent non-selected accessions. MGIDI, Multi-Trait Genotype–Ideotype Distance Index.

**Figure 8:**
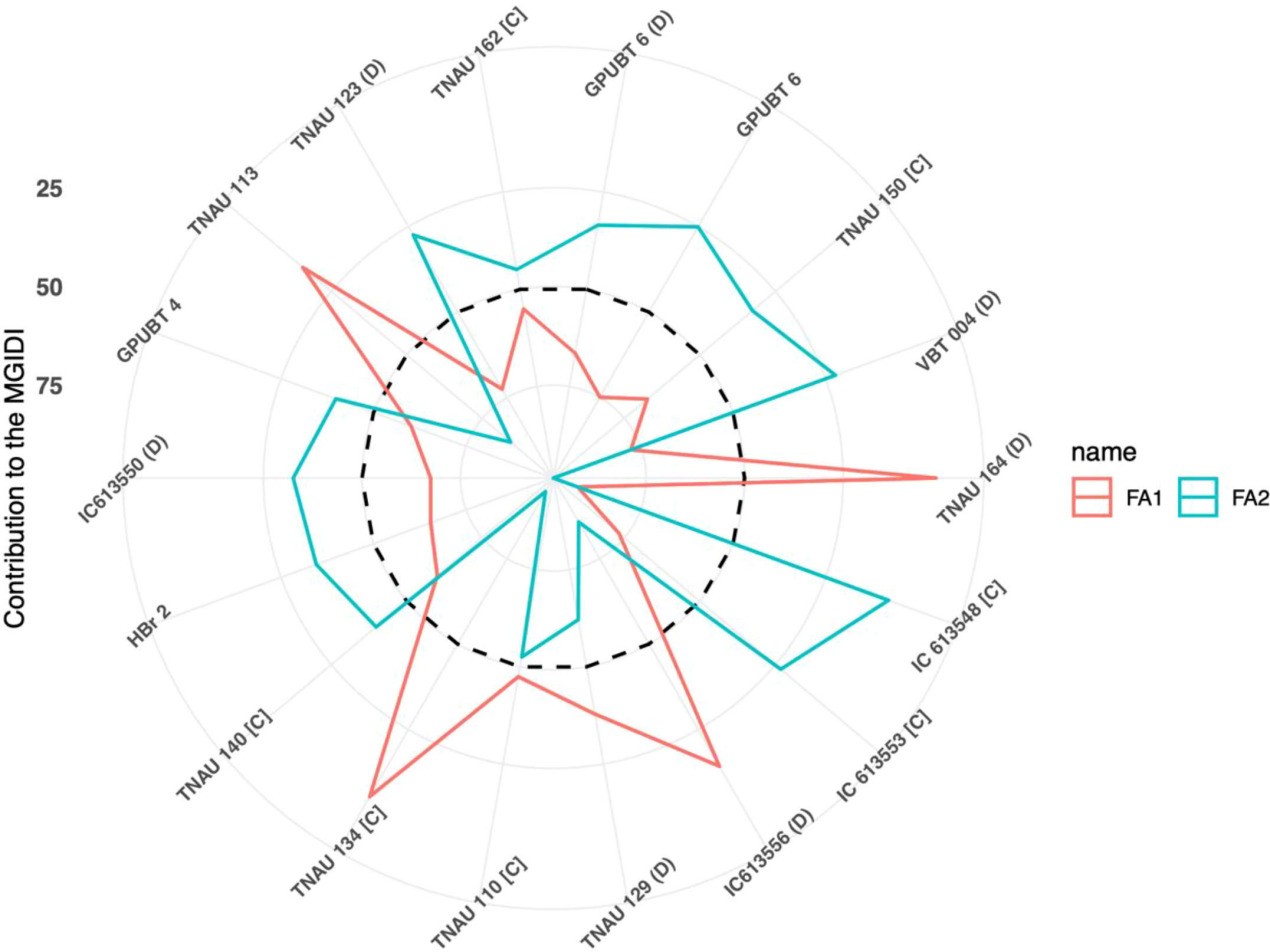
Radar plot representing strengths and weaknesses view of selected browntop millet (*Urochloa ramosa* L.) germplasm accessions based on the Multi-Trait Genotype–Ideotype Distance Index (MGIDI). The radar plot displays the contribution of the first two extracted factors (FA1 and FA2) to the MGIDI value of selected genotypes. FA1, explaining 33.12% of total variance, predominantly represents yield and its associated i.e., number of tillers per plant, panicles per plant, panicle length, and grain yield. FA2, accounting for 15.45% of variance, mainly represents biomass and maturity-related traits such as fodder yield, thousand seed weight, and days to maturity. The radial axis represents the percentage contribution of each factor to the MGIDI index. Lower contributions indicate strengths (traits closer to the ideotype), whereas higher contributions indicate weaknesses. The average factor contribution was represented in the form of dashed circular line.

The multi-trait genotype ideotype distance index was used to identify high-yielding genotypes by considering the pooled means of all 12 traits evaluated across both seasons. Based on the PCA loadings and exploratory factor analysis, two factors with eigenvalues greater than 1 were identified, collectively accounting for 48.57% of the total variability (**Table 7**). The MGIDI ranking identified 18 superior genotypes as indicated by their position above the selection threshold in the MGIDI plot (**Figure 7**), suggesting their suitability for further breeding and selection. Significant selection gains were observed for grain yield per plant (29.84%). Other traits showed selection gains, as follows: plant height (9.62%), fodder yield per plant (7.06%), number of tillers per plant (6.58%), panicle length (6.11%), and panicles per plant (4.91%). The genetic gain for plant height was 9.62%, whereas for days to flowering, it was a minimum of 1.79%, but in the opposite direction (**Table 8**). From the **Figure 8**, among the 18 identified genotypes TNAU 113, TNAU 134 [C], TNAU 164 [D], and IC613556 [D] demonstrated strengths related to factor 1, while the remaining genotypes showed strengths towards factor 2.

**Table 7:** Factor loadings, eigen values and variances explained and eigen values of traits considered for multi-trait genotype ideotype distance index (MGIDI).

S. no	Traits	FA1	FA2
1	DM	-0.001	-0.622
2	PHT	-0.69	-0.29
3	NTP	0.741	0.025
4	PPL	0.78	-0.155
5	PL	0.665	0.124
6	GYP	0.549	0.535
7	FYP	0.096	0.614
8	TW	0.03	0.57
9	Eigen values	2.649	1.236
10	Variance (%)	33.12	15.45
11	Accumulated (%)	33.12	48.57

DM, Days to maturity; PHT, Plant height (cm); NTP, Number of basal tillers per plant; PPL, Panicles per plant; PL, Panicle length (cm); GYP, Grain yield per plant (g); FYP, Fodder yield per plant (g); TW, Thousand seed weight (g).

**Table 8:** Selection gains for mean performance of browntop millet germplasm.

S.no	Var	Factor	Xo	Xs	SD	SD (%)	h2b	SG	SG (%)	Sense	Goal
1	PHT	FA1	97.38	108	10.65	10.94	0.88	9.37	9.62	decrease	No
2	NTP	FA1	9.12	9.77	0.64	7.06	0.74	0.48	5.22	increase	yes
3	PPL	FA1	9.57	10.18	0.61	6.38	0.81	0.49	5.16	increase	yes
4	PL	FA1	14.17	15.26	1.099	7.76	0.78	0.86	6.05	increase	yes
5	GYP	FA1	14.59	19.9	5.309	36.38	0.82	4.35	29.84	increase	yes
6	DM	FA2	91.02	92.24	1.22	1.34	0.98	1.20	1.31	decrease	No
7	FYP	FA2	91.11	99.8	8.695	9.54	0.93	8.09	8.88	increase	yes
8	TW	FA2	2.97	3.07	0.10	3.47	0.82	0.08	2.85	increase	yes

DM, Days to maturity; PHT, Plant height (cm); NTP, Number of basal tillers per plant; PPL, Panicles per plant; PL, Panicle length (cm); GYP, Grain yield per plant (g); FYP, Fodder yield per plant (g); TW, Thousand seed weight (g).

### 3.8 Trait specific sources

Based on the pooled Best Linear Unbiased Prediction (BLUP) means, trait-specific sources were identified for key traits such as days to maturity, grain yield per plant (g), fodder yield per plant (g), and 1000 seed weight (g). The top 10 performing accessions for these traits were identified as trait-specific sources. In addition to the pooled means, the performance of these accessions during the individual seasons is presented in **Table 9**. According to the pooled means, the crop duration of trait-specific sources identified for early maturity ranged from 84 to 87 days, which was consistent in 2023, and ranged from 84 to 89 days in 2024. The pooled grain yield per plant (g) of superior accessions ranged from 19.78 g to 24.85 g. Furthermore, the performance of these accessions in 2023 and 2024 exhibited variability, ranging from 18.33 to 22.91 g in 2023 and from 20.11 to 26.43 g in 2024, respectively. In addition to grain yield, trait-specific accessions for fodder yield were also identified, with pooled means ranging from 120.75 g to 153.03 g. The performance of these accessions in individual years ranged from 113.34 g to 160.41 g in 2023 and from 111.74 g to 141.79 g in 2024. The thousand seed weight of trait-specific accessions identified under pooled conditions ranged from 3.19 g to 3.51 g. The performance of these accessions in individual seasons ranged from 3.29 to 3.66 g in 2023 and from 3.46 to 3.71 g in 2024. All trait-specific sources identified for days to maturity, grain yield, and fodder yield were statistically superior to the trial mean across both years and in the pooled analysis. However, in the case of 1000 seed weight, only three accessions were statistically superior to the trial mean in both pooled and individual year analyses, while others were on par with the trial mean. The accession TNAU-134 [c] was superior for both early maturity and grain yield, TNAU-140 [c] for both grain and fodder yield, TNAU-150 [c] and GPUBT 6 [D] for grain yield and 1000 seed weight, and IC 613353 [C] for fodder yield and 1000 seed weight.

**Table 9:** Traits specific sources of browntop millet germplasm.

Genotype	Days to maturity			Grain yield per plant (g)			Fodder yield per plant (g)			Thousand seed weight (g)		
	Pooled	2023	2024	Pooled	2023	2024	Pooled	2023	2024	Pooled	2023	2024
TNAU 130 [D]	84*	84*	84*	13.39	11.11	15.83	81.23	84.74	77.20	3.01	3.18	3.30
KMBT 1	85*	84*	86*	7.73	6.83	9.21	76.56	78.99	74.91	2.95	3.12	3.24
TNAU 153 [D]	85*	84*	86*	13.49	13.70	13.35	77.91	80.65	75.58	3.05	3.19	3.34
TNAU 151 [C]	85*	85*	86*	14.25	15.38	13.07	65.03	62.98	68.83	2.71	2.89	3.04
TNAU 134 [C]	86*	85*	87*	22.12*	20.82*	22.76*	94.48	85.66	102.55	3.18*	3.37*	3.40
TNAU 130	86*	85*	87*	11.27	10.50	12.34	65.53	65.84	66.36	2.74	2.93	3.05
TNAU 134 [D]	87*	86*	88*	16.07	15.62	16.33	114.96*	118.83*	110.00*	3.15	3.29	3.43
TNAU 126 [C]	87*	87*	88*	18.12*	17.25*	18.66	82.59	80.55	84.01	3.03	3.17	3.32
TNAU 140	87*	87*	88*	11.63	11.83	11.66	61.81	62.20	63.41	3.15	3.33	3.40
Ganiger collection	87*	86*	89*	16.71	15.37	17.89	99.54	99.12	99.10	3.08	3.25	3.35
TNAU 140 [C]	91	90	92	19.78*	18.33*	20.81*	141.78*	147.62*	133.17*	3.16	3.28	3.47
IC 613548 [C]	92	91	93	19.83*	18.92*	20.28*	115.45*	121.88*	106.89*	2.97	3.11	3.30
TNAU 150 [C]	91	91	92	20.21*	19.79*	20.11*	111.49*	112.26*	110.27*	3.25*	3.42*	**3.49**
TNAU 164 [D]	92	91	93	21.86*	17.41*	25.78*	97.16	94.44	100.01	3.18*	3.28	3.49
IC 613553 [C]	93	91	94	22.39*	18.02*	26.18*	79.46	79.42	80.59	3.08	3.20	3.40
TNAU 110 [C]	91	90	92	22.65*	19.83*	24.81*	95.75	98.38	93.47	2.95	3.18	3.18
TNAU 129 [D]	92	90	93	23.03*	18.99*	26.43*	80.02	79.82	81.83	2.99	3.28	3.16
GPUBT 6 [D]	92	91	93	24.18*	22.63*	24.90*	81.70	81.52	81.92	3.20*	3.36*	**3.46**
VBT 004 [D]	91	91	92	24.85*	22.91*	25.91*	107.17*	111.06*	102.27	3.06	3.18	3.37
TNAU 144 [C]	91	90	92	15.41	17.67	12.95	120.75*	129.57*	111.80*	2.84	2.95	3.20
TNAU 18 [C]	89	88	91	13.81	12.44	15.23	121.19*	113.34*	127.27*	3.10	3.26	3.37
TNAU 118	88	87	89	16.33	14.53	18.01	122.93*	131.69*	111.74*	2.83	2.98	3.16
TNBr 012	89	88	90	15.05	15.25	14.77	123.33*	128.77*	116.30*	3.13	3.27	3.41
IC 613353 [C]	94	92	95	9.74	9.79	10.06	126.63*	137.20*	114.58*	3.24*	3.36*	**3.51**
TNAU 122 [C]	91	90	93	16.84	15.18	18.34	130.18*	138.91*	118.65*	3.09	3.23	3.38
IC618552	93	92	94	14.51	13.78	15.25	130.76*	138.91*	119.77*	3.08	3.22	3.37
TNAU 158 [C]	91	90	92	12.40	11.11	13.90	132.03*	137.90*	123.31*	2.93	3.10	3.22
TNAU 136 [D]	91	89	92	16.54	13.60	19.37*	153.03*	160.41*	141.79*	2.80	2.94	3.13
VBTG 5 [D]	91	90	92	15.02	15.51	14.45	80.94	66.17	94.66	3.19*	**3.31**	**3.49**
VBTG 9 [D]	96	94	97	11.18	10.82	11.83	69.98	70.87	70.70	3.23*	**3.33**	3.53*
IC 613555 [D]	87	87	88	14.71	13.44	15.98	61.96	62.59	63.32	3.23*	3.35*	3.52*
HBr 2	94	93	95	18.09*	16.28	19.62*	114.43*	119.63*	108.09*	3.24*	**3.29**	3.58*
IC 613562 [C]	91	90	92	10.47	9.63	11.65	104.12	99.49	107.95	3.26*	3.44*	**3.48**
IC 617959 [C]	91	90	92	7.61	7.12	8.71	81.11	82.12	81.15	3.29*	3.39*	3.59*
VBTG 7 [D]	93	92	93	13.47	10.26	16.87	109.60*	110.51*	108.32*	3.51*	3.66*	3.71*
**Trail mean**	91	90	92	14.59	13.62	15.56	91.11	91.01	91.20	3.20	3.12	3.27
**Trail Range**	84-98	84-97	84-99	6.70 - 24.85	6.50-22.91	7.30-26.43	52.94 - 153.03	45.76-160.41	59.59-141.79	2.43 - 3.51	2.51 - 3.66	2.84- 3.71
**LSD (5%)**	1.41	1.53	1.28	3.33	3.20	3.46	3.33	3.20	3.46	0.21	0.22	0.25
**CV (%)**	0.78	0.86	0.70	11.51	11.86	11.23	11.51	11.86	11.23	3.33	3.48	3.79

Bold values indicate trait specific accessions for that trait and * indicates the statistical superiority over trail mean at 5% level of significance.

## 4 Discussion

Browntop millet is one of the important small millets known for its high nutritional value, climate resilience, and superior market price relative to other small millets. However, systematic germplasm characterization and utilization remain limited., despite its significance. As of now, only two cultivars have been made available for commercial cultivation (Nandini et al., 2025a). Therefore, it is essential to expedite research aimed at developing high-yielding cultivars of this crop. Germplasm serves as the fundamental raw material for breeding programs. Significant variations are present in browntop millet germplasm, and efforts to utilize existing diversity have been limited. This study aims to compile all available *Urochloa ramose* germplasm from the National Active Germplasm Site (NAGS) of small millet in India and to characterize them based on qualitative and quantitative traits. Assessing germplasm resources and identifying trait-specific sources is crucial for revealing genetic diversity and exploring untapped genetic potential. This may also improve the utility of current germplasm resources for crop improvement. Subsequent evaluation of germplasm and direct selection for superior trait performance across various environments has resulted in the release of new cultivars in numerous small millet crops (Vetriventhan et al., 2020). The effects of pureline selection in brown top millet, particularly from the germplasm IC617960 and browntop Kolour, are reflected in the development of cultivars like GPUBT 2 and Hbr-2. This illustrates the variability present in the browntop millet germplasm conserved throughout India, facilitating the assessment of diversity and the identification of trait-specific accessions related to agro-morphological characteristics.

The examined genotypes exhibited significant variations in qualitative traits such as inflorescence shape, inflorescence compactness, and panicle pubescence. In terms of inflorescence shape, compact types were observed more frequently than open types, and genotypes exhibiting pubescence were also more prevalent. The open-type panicle genotypes exhibit greater resistance to shattering compared to closed type panicles. The frequency of green types was higher concerning pigmentation on nodes, leaf sheath, and auricle. Nonetheless, pigmentation was observed in 10-13% of the genotypes. These pigmented genotypes are beneficial in hybridization programs due to the highly self-pollinated nature of browntop millet (Nagaraja et al., 2023; Nandini et al., 2025b). In the process of artificial hybridization, pigmented plants can be used as male parents, facilitating the identification of the authentic F_1_ hybrids (Nandini et al., 2025b, Nandini et al., 2025a). Therefore, the pigmented types identified in this study can serve as effective phenotypic markers for hybridization in crop improvement programs, while morphological characteristics provide a basis for the identification, conservation, and accurate description of landraces. Similar morphological variability has been reported in other small millet crops and is considered an indicator of genetic diversity within traditional germplasm collections (Goron and Raizada, 2015; Fukunaga et al., 2024).

The assessment of quantitative traits in browntop millet revealed significant genotypic variance components for all morphological traits across the individual years as well as in the pooled analysis, indicating considerable genetic variability among the studied genotypes. The presence of high heritability coupled with high genetic advance in several traits including grain yield suggests that additive gene action plays a crucial role in inheritance. Traits governed by additive gene effects respond effectively to phenotypic selection and therefore can be improved through early generation selection in breeding programs. This trend has also been observed in various millets, where yield and yield-related traits with high heritability have been successfully improved using direct selection methods (Yadav et al., 2021; Anuradha et al., 2024). The Wald statistic demonstrates a substantial seasonal effect on trait expression. The non-significant genotype × season interaction, except for grain and fodder yield, indicates a consistent environmental effect across genotypes and comparable trait expression responses. The significant variation in the mean and range of essential traits, including flowering time, maturity, plant height, and yield components, highlights the diverse genetic foundation and potential for enhancement of the examined germplasm accessions. The 1000-seed weight of browntop millet in this study was 3.2 g, which exceeds that of finger millet (2.6 g) (Backiyalakshmi et al., 2023) and little millet (2.2 g), yet is lower than that of kodo millet (4.8 g) (Vetriventhan and Upadhyaya, 2019).

The correlation and path coefficient analysis revealed a complex network of associations among the examined traits, indicating that grain yield in browntop millet is regulated by multiple genes and affected by various yield-contributing factors. Grain yield per plant showed significant positive correlations with plant height, number of tillers per plant, number of panicles per plant, panicle length, thousand seed weight, and fodder yield per plant. This indicates that these traits are crucial in influencing yield potential, and their concurrent improvement could increase grain yield. A study on browntop millet indicated that grain yield per plant significantly correlates positively with plant height, number of panicles per plant, panicle length, peduncle length, flag leaf width, and flag leaf length (Rahul et al., 2024). In foxtail millet, a positive correlation was identified between grain yield and both the number of tillers per plant and panicle length (Nandini et al., 2018). Studies on barnyard millet revealed a significant positive association between grain yield per plant and several traits, including days to 50% flowering, plant height, number of productive tillers, days to maturity, and straw weight (Nandini et al., 2020). The non-significant positive correlation between yield and days to flowering, days to maturity, flag leaf length, and flag leaf and panicle width indicates a limited direct effect on yield, while suggesting possible indirect contributions under specific environmental conditions. The positive correlations between plant height, tiller count per plant, panicle count per plant, and panicle length suggest a coordinated growth pattern in browntop millet, wherein enhanced vegetative growth facilitates increased reproductive output. Similar observations were noted in other small millet crops, including barnyard millet and proso millet, where a positive correlation was found between grain yield and vegetative traits such as plant height and stover yield per plant (Nandini et al., 2020; Vetriventhan et al., 2024). Additionally, in proso millet, grain yield was positively associated with plant height, leaf number, and basal tillers (Calamai et al., 2020). Path coefficient analysis indicated that plant height, number of tillers per plant, number of panicles per plant, panicle length, and fodder yield per plant exerted significant positive direct effects on grain yield, suggesting true cause-and-effect relationships rather than mere indirect associations, these characters may serve as reliable selection criteria in breeding programs aimed at improving yield potential. The moderate intercorrelations observed among independent variables did not indicate severe multicollinearity, suggesting that the estimated direct effects were stable and interpretable. A high residual value suggests the influence of various other traits that significantly contribute to grain yield. Therefore, it is crucial to prioritize the identification of these traits and their incorporation into the list of trait descriptors in future development efforts. The results indicate that the selection of genotypes characterized by tall stature, high tillering, elongated panicles, and substantial fodder yield potential can significantly enhance grain yield in browntop millet. These traits serve as reliable selection criteria for dual-purpose breeding programs (grain and fodder) in dryland environments.

Hierarchical clustering of the four cluster groups demonstrated notable differences among the genotypes across the various clusters. Germplasm collected from Andhra Pradesh, Tamil Nadu, and Karnataka exhibited clustering of certain accessions from different states, suggesting genetic similarities. Cluster 4 demonstrated elevated cluster means for traits including grain yield per plant, plant height, and fodder yield per plant, all of which exhibited a significant positive correlation. This indicates that the genotypes within this cluster should be prioritized for the selection of these traits. The breeding program for dwarf cultivars should prioritize cluster 2, while exercising caution due to the potential yield penalty linked to this positive correlation. Despite no significant association between 1000-seed weight and grain yield per plant, genotypes in cluster 1, although lower yielding than those in cluster 4, performed similarly in 1000-seed weight. This indicates that choosing genotypes from these two clusters for hybridization may result in the development of dwarf cultivars characterized by bold seeds and higher yields. Principal component analysis (PCA) serves as a robust multivariate method for identifying the key traits that contribute to genetic divergence in crop germplasm. This study found that the first five principal components (PCs) with eigenvalues exceeding one accounted for 68.66% of the total variability, suggesting a substantial genetic diversity among the assessed browntop millet accessions. The initial five principal components, exhibiting eigenvalues exceeding one, accounted for contributions of 24.98%, 16.72%, 9.52%, 8.75%, and 8.69%, underscoring their significance in representing the variability of the assessed traits. The acute angle among the traits of grain yield, plant height, and panicle length suggests a positive correlation, as evidenced by the correlation analysis. The research conducted by (Rahul et al., 2024) identified grain yield per plant, panicle width, and flag leaf length as the primary factors contributing to the variation observed in the principal component analysis of browntop millet. Research on foxtail millet revealed that traits including days to 50% flowering, plant height, peduncle length, flag leaf length, and flag leaf width significantly contributed to overall variability (Nandini et al., 2018). In barnyard millet, the primary contributors to variation include days to 50% flowering, plant height, days to maturity, and straw weight (Nandini et al., 2020). In proso millet first three principal components explained 57.63% of variance (Lv et al., 2025).

The identification of trait-specific sources is essential for the effectiveness of breeding programs. The main emphasis has been on traits including grain and fodder, days to maturity for early duration variety development, and 1000-seed weight for bold-seeded varieties. The pooled means of the identified early maturity germplasm ranged from 84 to 87 days, significantly less than the maturity durations of the released varieties GPUBT 2 and HBr2, which are 93 and 94 days, respectively. The yields of these varieties were recorded at 16.98 and 18.09 g/plant, respectively, while all identified trait-specific sources demonstrated a mean yield exceeding 19, with values ranging from 19.78 to 24.85 g/plant. The accessions GPUBT 6 [D] and VBT 004 [D] yielded 24.18 and 24.85 g/plant, respectively, indicating a yield advantage of approximately 25% compared to the germplasm released as varieties. The pooled performance of the trait-specific germplasm for 100-seed weight ranged from 3.19 to 3.51 g, comparable to HBr2 at 3.24 g. Notably, the accession VBTG 7 [D] exhibited an approximately 8% higher 1000-seed weight at 3.51 g. The identified accessions for various traits include TNAU-134 [c] for early maturity and high grain yield, TNAU-140 [c] as dual-purpose for grain and fodder yield, TNAU-150 [c] and GPUBT 6 [D] for grain yield and 1000-seed weight, and IC 613353 [C] for fodder yield and 1000-seedweight. Comparable studies focusing on the identification of trait-specific germplasm accessions in other small millets have been conducted by (Vetriventhan et al., 2024) and (Reddy et al., 2025), underscoring the importance of systematic germplasm characterization for harnessing genetic diversity and identifying superior donor lines for crop improvement.

The multi-trait genotype ideotypes index is an approach utilizing Euclidean distance-based factor analysis to identify the best-performing genotypes according to MGIDI values. This method has been effectively applied in several key cereals, including rice (Al Mamun et al., 2024; Feizi et al., 2025), wheat (Nardino et al., 2022) and maize (Singamsetti et al., 2023) as well as in finger millet (Ghetiya et al., 2025) and foxtail millet (Chaturvedi and Rao, 2025). Genotypes exhibiting low MGIDI values demonstrated superior performance for the specified traits and identified 18 genotypes that corresponded with the objectives of the breeding program, primarily emphasizing high yield and related traits. Eleven genotypes GPUBT 6 [D], VBT 004 [D], TNAU 164 [D], IC 613548 [C], IC 613553 [C], TNAU 129 [D], TNAU 110 [C], TNAU 134 [C], TNAU 140 [C], HBr 2, and TNAU 150 [C] are consistently identified as high performers according to both MGIDI and trait-specific evaluations. Research on little millet, proso millet, and kodo millet has revealed trait-specific accessions related to grain yield and nutritional characteristics through multilocation germplasm evaluations (Vetriventhan and Upadhyaya, 2018, Vetriventhan and Upadhyaya, 2019; Vetriventhan et al., 2021). The selection goals were met for all traits assessed, with the exception of earliness and dwarf plant phenotype. No significant correlation was observed between flowering duration, maturity duration, and yield. Therefore, selecting high-yield performers with early maturity is essential, as the maturity duration range (86–93 days) of the identified high-yield varieties is still shorter than that of the released varieties GPUBT 2 and HBr2, which have maturity durations of 93 and 94 days, respectively. A highly significant selection was noted for grain yield (30.29%). The evaluation of identified germplasm accessions across multiple locations and seasons can aid in identifying the highest-performing lines for potential variety release.

## 5 Conclusion

In conclusion, comprehensive characterization of the available browntop millet germplasm revealed substantial genetic variability for grain yield and key agronomic traits, with high heritability estimates supporting the effectiveness of direct phenotypic selection in early breeding stages. Multivariate analyses, including PCA, hierarchical clustering, and the MGIDI index, effectively differentiated diverse groups and identified TNAU-134, TNAU-140, TNAU-150, GPUBT-6, and IC 613353 as promising donor accessions for enhancing yield, early maturity, and dual-purpose performance. These findings provide a strong foundation for strategic hybridization and cultivar development aimed at improving productivity and climate resilience. The selected accessions warrant multi-environment and multi-season evaluation to confirm yield stability and agronomic superiority prior to potential varietal release. Integration of molecular characterization in future studies will further complement phenotypic evaluation and strengthen precision breeding efforts in browntop millet.

## Data Availability

The original contributions presented in the study are included in the article/**Supplementary Material**. Further inquiries can be directed to the corresponding authors.

## Supplementary material

Supplementary Table 1 List of Browntop millet germplasms and their site of collection used for characterization. [C] represents the type chaduru korle- open type panicle, [D] represents dundu korle- compact closed type panicle.

Supplementary Table 2 Direct and indirect effects of various traits on grain yield of Browntop millet germplasm evaluated. DFF, Days to Flowering; DM, Days to maturity; PHT, Plant height (cm); FLL, Flag leaf length (cm); FLW, Flag leaf width (cm); NTP, Number of basal tillers per plant; PPL, Panicles per plant; PL, Panicle length (cm); PW, Panicle width (cm); GYP, Grain yield per plant (g); FYP, Fodder yield per plant (g); TW, Thousand seed weight (g).
