# Supplementary material for: Trait phenotyping and identification of trait-specific donor genotypes for agronomic improvement and ideotype breeding in browntop millet (Urochloa ramosa L.)
Source: Front Plant Sci. 2026 Apr 13;17:1756143. doi: 10.3389/fpls.2026.1756143 (PMC13111081; doi:10.3389/fpls.2026.1756143)
Supplement: Supplementary Table 1 — List of Browntop millet germplasms and their site of collection used for characterization. [C] represents the type chaduru korle- open type panicle, [D] represents dundu korle- compact closed type panicle. [file DataSheet1.pdf]

Table S1: List of Browntop millet germplasms and their site of collection used for characterization

Sl. No.	Genotype	State of collection	Sl. No.	Genotype	State of collection	Sl. No.	Genotype	State of collection
1	IC 617953 (D)	Karnataka	41	TNAU 126 (D)	Tamil Nadu	81	TNAU 133 [C]	Tamil Nadu
2	IC613556 (D)	Karnataka	42	TNAU 171 [C]	Tamil Nadu	82	TNAU 114 [C]	Tamil Nadu
3	IC 613546	Karnataka	43	TNAU 140 [C]	Tamil Nadu	83	TNAU 158 [C]	Tamil Nadu
4	GPUBT 6	Karnataka	44	TNAU 128 (D)	Tamil Nadu	84	TNAU 126 [C]	Tamil Nadu
5	IC613557 [C]	Karnataka	45	TNAU 115 [D]	Tamil Nadu	85	TNAU 131 (D)	Tamil Nadu
6	Ganiger collection	Karnataka	46	IC613558	Tamil Nadu	86	TNAU 136 (D)	Tamil Nadu
7	GPUBT 6 (D)	Karnataka	47	IC618552	Tamil Nadu	87	TNAU 155	Tamil Nadu
8	IC 613548 [C]	Karnataka	48	TNAU 130 (D)	Tamil Nadu	88	TNAU 117 [C]	Tamil Nadu
9	Bijapur Collection	Karnataka	49	TNAU 118	Tamil Nadu	89	TNAU 125 [C]	Tamil Nadu
10	IC 613554 (D)	Karnataka	50	TNAU 122 [C]	Tamil Nadu	90	TNAU 155 [C]	Tamil Nadu
11	KMBT 1	Karnataka	51	TNAU 129	Tamil Nadu	91	TNAU 154 (D)	Tamil Nadu
12	IC 613353 [C]	Karnataka	52	TNAU 163 [C]	Tamil Nadu	92	TNAU 128 [C]	Tamil Nadu
13	IC 613549	Karnataka	53	TNAU 137 [D]	Tamil Nadu	93	TNAU 153 (D)	Tamil Nadu
14	IC 613562 [C]	Karnataka	54	TNAU 147 [C]	Tamil Nadu	94	TNAU 134 [C]	Tamil Nadu
15	IC 617961 [C]	Karnataka	55	TNAU 145 [C]	Tamil Nadu	95	TNAU 164 [C]	Tamil Nadu
16	IC 613563 (D)	Karnataka	56	TNAU 161 [C]	Tamil Nadu	96	TNAU 120 (C)	Tamil Nadu
17	IC 613554 [C]	Karnataka	57	TNAU 134 (D)	Tamil Nadu	97	TNAU 128 [C]	Tamil Nadu
18	IC617957 [C]	Karnataka	58	TNAU 18 [C]	Tamil Nadu	98	TNAU 151 (D)	Tamil Nadu
19	IC613559 (D)	Karnataka	59	TNAU 124 (D)	Tamil Nadu	99	TNAU 120 (D)	Tamil Nadu
20	IC613550 (D)	Karnataka	60	TNAU 133	Tamil Nadu	100	TNAU 140	Tamil Nadu
21	IC 613552 (D)	Karnataka	61	TNAU 151 [C]	Tamil Nadu	101	TNAU 130 [C]	Tamil Nadu
22	IC 613553 (D)	Karnataka	62	TNAU 125 (D)	Tamil Nadu	102	TNAU 142	Tamil Nadu
23	IC 613550 [C]	Karnataka	63	TNAU 110 [C]	Tamil Nadu	103	TNAU 159	Tamil Nadu
24	IC 617956 [C]	Karnataka	64	TNAU 113 [C]	Tamil Nadu	104	TNBr 012	Tamil Nadu
25	IC 617959 [C]	Karnataka	65	TNAU 149 [C]	Tamil Nadu	105	TNAU 109	Tamil Nadu
26	IC 617954	Karnataka	66	TNAU 164 (D)	Tamil Nadu	106	VBT 001 (D)	Andhra Pradesh
27	IC 613553 [C]	Karnataka	67	TNAU 129 (D)	Tamil Nadu	107	VBTG 5	Andhra Pradesh
28	GPUBT 2	Karnataka	68	TNAU 131 [C]	Tamil Nadu	108	VBTG 1	Andhra Pradesh
29	IC 613555 (D)	Karnataka	69	TNAU 166 [C]	Tamil Nadu	109	VBTG 10 (D)	Andhra Pradesh
30	GPUBT 3	Karnataka	70	TNAU 161 (D)	Tamil Nadu	110	VBTG 5 (D)	Andhra Pradesh
31	GPUBT 1	Karnataka	71	TNAU 162 [C]	Tamil Nadu	111	VBTG 7 [C]	Andhra Pradesh
32	GPUBT 4	Karnataka	72	TNAU 169 [C]	Tamil Nadu	112	VBTG 3 [C]	Andhra Pradesh
33	GPUBT 7	Karnataka	73	TNAU 144 [C]	Tamil Nadu	113	VBTG 8 (D)	Andhra Pradesh
34	HBr 2	Karnataka	74	TNAU 152 (D)	Tamil Nadu	114	VBTG 3	Andhra Pradesh
35	TNAU 138 [C]	Tamil Nadu	75	TNAU 150 [C]	Tamil Nadu	115	VBTG 7 (D)	Andhra Pradesh
36	TNAU 135 (D)	Tamil Nadu	76	TNAU 136 [C]	Tamil Nadu	116	VBTG 9 (D)	Andhra Pradesh
37	TNAU 123 (D)	Tamil Nadu	77	TNAU 154	Tamil Nadu	117	VBT 004 (D)	Andhra Pradesh
38	TNAU 135 [c]	Tamil Nadu	78	TNAU 113	Tamil Nadu	118	VBTG 6 [C]	Andhra Pradesh
39	TNAU 116	Tamil Nadu	79	TNAU 142 [C]	Tamil Nadu	119	VBTG 1 (D)	Andhra Pradesh
40	TNAU 143	Tamil Nadu	80	TNAU 160	Tamil Nadu	120	VBT001 [C]	Andhra Pradesh
						121	BTMNDL-3	Andhra Pradesh

Table S2. Direct and indirect effects of various traits on grain yield of Browntop millet germplasm evaluated.

	DFF	DM	PHT	FLL	FLW	NTP	PPL	PL	PW	FYP	TW	GY
**DFF**	**-13.355**	13.349	0.015	0.022	0.012	0.005	0.007	0.027	0.010	0.072	0.016	**0.11**
**DM**	-13.355	**13.349**	0.015	0.020	0.012	0.002	0.005	0.025	0.010	0.061	0.016	**0.11**
**PHT**	-2.003	2.002	**0.102**	0.041	0.010	0.079	0.077	0.076	-0.014	0.089	0.011	**0.40****
**FLL**	-1.336	1.201	0.019	**0.217**	0.007	0.009	0.018	0.041	-0.028	0.017	0.003	**0.14**
**FLW**	-3.205	3.070	0.019	0.028	**0.052**	0.014	0.002	0.005	-0.003	0.045	0.014	**-0.04**
**NTP**	-0.267	0.133	0.035	0.009	0.003	**0.232**	0.116	0.047	-0.048	0.061	0.019	**0.45****
**PPL**	-0.401	0.267	0.035	0.017	0.001	0.119	**0.227**	0.052	-0.050	0.022	0.001	**0.27****
**PL**	-2.270	2.136	0.049	0.057	0.002	0.070	0.075	**0.158**	-0.017	0.050	0.002	**0.23***
**PW**	0.801	-0.801	0.008	0.035	0.001	0.065	0.066	0.016	**-0.172**	0.072	0.009	**0.09**
**FYP**	-1.736	1.468	0.016	0.007	0.004	0.026	0.009	0.014	-0.022	**0.557**	0.008	**0.30****
**TW**	-2.137	2.136	0.011	0.007	0.007	0.044	0.002	0.003	-0.015	0.045	**0.097**	**0.14**

Note: DFF- Days to Flowering,DM-Days to maturity, PHT- Plant height (cm), FLL- Flag leaf length (cm), FLW- Flag leaf width (cm), NTP- Number of basal tillers per plant, PPL- Panicles per plant, PL- Panicle length (cm), PW- Panicle width (cm), GYP- Grain yield per plant (g), FYP- Fodder yield per plant (g), TW- Thousand seed weight (g)
